# The CAMI-score: A Novel Tool derived From CAMI Registry to Predict In-hospital Death among Acute Myocardial Infarction Patients

**DOI:** 10.1038/s41598-018-26861-z

**Published:** 2018-06-13

**Authors:** Chenxi Song, Rui Fu, Kefei Dou, Jingang Yang, Haiyan Xu, Xiaojin Gao, Wei Li, Guofeng Gao, Zhiyong Zhao, Jia Liu, Yuejin Yang

**Affiliations:** Fuwai Hospital, National Center for Cardiovascular Diseases, Chinese Academy of Medical Sciences and Peking Union Medical College, Beijing, China

## Abstract

Risk stratification of patients with acute myocardial infarction (AMI) is of clinical significance. Although there are many existing risk scores, periodic update is required to reflect contemporary patient profile and management. The present study aims to develop a risk model to predict in-hospital death among contemporary AMI patients as soon as possible after admission. We included 23417 AMI patients from China Acute Myocardial Infarction (CAMI) registry from January 2013 to September 2014 and extracted relevant data. Patients were divided chronologically into a derivation cohort (n = 17563) to establish the multivariable logistic regression model and a validation cohort (n = 5854) to validate the risk score. Sixteen variables were identified as independent predictors of in-hospital death and were used to establish CAMI risk model and score: age, gender, body mass index, systolic blood pressure, heart rate, creatinine level, white blood cell count, serum potassium, serum sodium, ST-segment elevation on ECG, anterior wall involvement, cardiac arrest, Killip classification, medical history of hypertension, medical history of hyperlipidemia and smoking status. Area under curve value of CAMI risk model was 0.83 within the derivation cohort and 0.84 within the validation cohort. We developed and validated a risk score to predict in-hospital death risk among contemporary AMI patients.

## Introduction

Ischemic heart disease has become the leading contributor of disease burden worldwide^1^. Acute myocardial infarction (AMI) is the most severe manifestation of ischemic heart disease. In the United States, approximately 750000 individuals suffer from first or recurrent MI every year^2^. In Europe, the 30-day case-fatality rate ranged from 4.5% in Sweden and 15.4%in Latvia^3^. In China, an increase in AMI mortality rate was observed and the rate was over 50 per 100000 population in 2014^4^.

Considerable variability exists among patients with AMI and many factors have an impact on an individual’s prognosis. Careful risk stratification is of clinical significance, as it informs decisions regarding treatment strategies as well as triage among alternative levels of care and provides an opportunity to estimate patient’s prognosis. Guidelines from both the American College of Cardiology/American Heart Association^2,5^ and the European Society of Cardiology^6^ recommended that the most appropriate pharmacological and interventional management should be determined after comprehensive risk assessment.

Many risk models of in-hospital mortality have been developed among patients with acute coronary syndrome^7–11^. Among these scores, The Thrombolysis in Myocardial Infarction(TIMI) score and the Global Registry in Acute Coronary Events (GRACE) score are the most commonly used and are recommended in the guideline^6^. However, both scores were developed when patient characteristics and management differed significantly from now, and few participants were from Asia. Therefore, it is necessary to update the existing models and the purpose of our study is to develop a multivariable logistic regression model to predict in-hospital mortality risk among patients with AMI.

## Methods

### CAMI registry

Details of China AMI (CAMI) registry design and method were described previously^12^. Briefly, CAMI registry was a prospective, multicenter observational registry conducted in China, which included patients with AMI and collected data on patients’ demographics, clinical presentation, initial medical contact, medical history and risk factors, treatment and clinical outcomes. Data were collected at each participating site by trained clinical cardiologists using electronic clinical reporting form. A total of 108 hospitals with different levels covering a broad geographic region participated in the project, which assured a good representation of AMI patients from China. The CAMI registry was registered on www.Clinicaltrials.gov (registration number: NCT01874691).

Our study was approved by the institutional review board central committee at Fuwai Hospital, NCCD of China. Written informed consent was obtained from eligible patients before registration. We confirmed that all methods were performed in accordance with the relevant guidelines.

### Study population

All patients enrolled in CAMI registry were included in our study. Eligible patients were diagnosed with AMI including ST-segment elevation myocardial infarction (STEMI) and non–ST elevation myocardial infarction (NSTEMI) in accordance with the third universal definition of myocardial infarction^13^. AMI classified as type 1, 2, 3, 4b and 4c were included in CAMI registry^12^. Type 4a and type 5 were not eligible for the CAMI registry. We excluded those patients with missing or invalid data on age, BMI, admission diagnosis and in-hospital outcome.

### Outcome measurement and clinical definition

The primary endpoint was all-cause in-hospital death defined as cardiac or non-cardiac death during hospitalization. Medical history and vital signs were determined at the time of first hospital presentation. Standard definition of the history and physical examination elements were well described in the ACC/AHA Task Force on clinical Data Standards and NCDR-ACTION-GWTG element dictionary. ECG and echocardiogram were interpreted locally.

### Statistical analysis

Baseline continuous variables were presented as mean ± SD or median (25th and 75th percentiles), and categorical variables were presented as counts and percentages. We used Student t tests to compare the continuous variables between in-hospital deaths and survivors, and chi-square tests to compare categorical variables. Univariate logistic regression was performed to examine the association between individual baseline variable and in-hospital mortality, which was described as odds ratio (OR) and 95% confidence interval (CI). All variables that achieved a significance level of P ≤ 0.25 were selected to fit the multivariable logistic regression model. Stepwise selection process was used to identify independent predictors of in-hospital death.

After selection, those variables with P < 0.05 were retained in the final model. A simplified risk score was developed for clinical practice by attributing integer numbers to these variables. The variable with the smallest estimated coefficient (reference variable) was attributed 1 point. The scores of other variables were determined by dividing their estimated coefficients by the coefficient of the reference variable^14^. We used area under curve (AUC) value and Hosmer-Lemeshow (HL) goodness-of-fit test to assess discrimination and calibrationability of the model respectively.

### Data availability statement

The data used in our study was from CAMI registry dataset and is not publicly available but is available from corresponding author on reasonable request.

## Results

### Baseline characteristics

**Figure 1:**
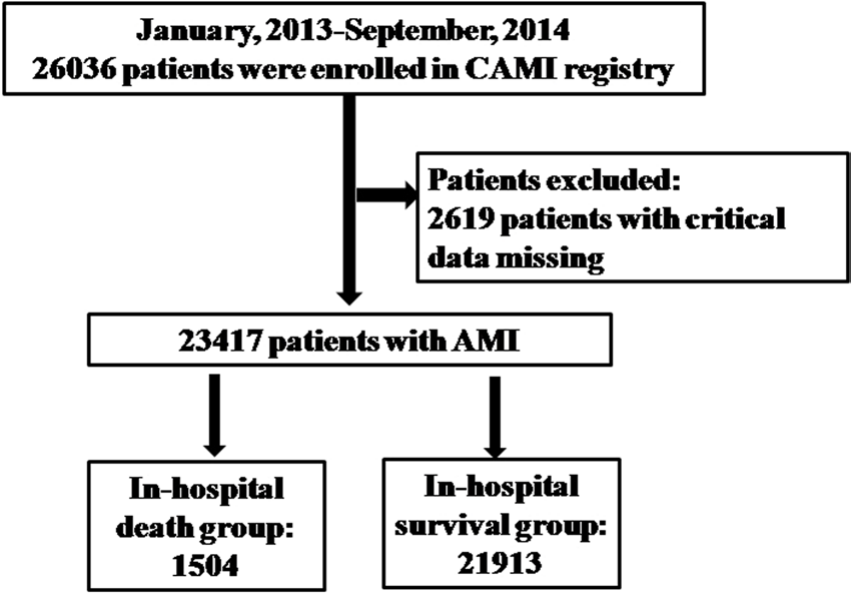
Study flow chart. From January 2013 to September 2014, a total of 26036 patients were enrolled in CAMI registry. After excluding 2619 patients due to critical data missing, we finally included 23417 AMI patients. A total of 1504 patients died during hospitalization.

From January 1^st^ 2013 to September 30^th^ 2014, a total of 26036 patients were enrolled in CAMI registry. We excluded 2619 patients with incomplete data on age, BMI, diagnosis on admission, in-hospital outcome and finally included 23417 patients in our study. There were 1504 (6.4%) patients died in the study sample (Fig. 1). Baseline characteristics are shown in Table 1. Compared with survivors, patients who died were older, more often female and had higher BMI. Proportion of cardiac arrest and higher Killip classification were higher among deaths vs. survivors. Patients who died also had more comorbidities.

**Table 1:** Baseline characteristics between in-hospital deaths vs. survivors.

Variables	In-hospital deaths N = 1504	In-hospital survivors N = 21913	P value
Age (years)	72.47 (64.21, 78.99)	62.41 (53.36, 71.57)	<0.001
Female (%)	42.2	24.4	<0.001
BMI (kg/m^2^)	23.19 (21.37, 25.14)	24.03 (22.19, 25.95)	<0.001
Chest pain (%)	71.8	74.2	0.0704
ST-segment elevation (%)	72.6	68.6	0.0012
Anterior wall involvement (%)	56.3	47.7	<0.001
SBP (mmHg)	116.00 (99.00, 135.00)	129.00 (112.00, 145.00)	<0.001
HR (bpm)	86.00 (70.00, 102.00)	76.00 (66.00, 87.00)	<0.001
Fatal arrhythmia (%)	16.3	6.5	<0.001
Cardiac arrest (%)	5.4	0.9	<0.001
Killip classification (%)			<0.001
I	44.6	76.8	
II	22.2	16.1	
III	12.1	4.3	
IV	21.1	2.8	
Medical history (%)			
Hypertension	2.9	1.4	<0.001
Hyperlipidemia	4.1	6.8	<0.001
Diabetes	23.2	18.7	<0.001
Premature family CAD	1.5	3.5	<0.001
MI	9.6	7	<0.001
PCI	3.4	4.7	0.0136
CABG	0.6	0.4	0.4913
Heart failure	6.5	2.1	<0.001
PAD	0.7	0.6	0.6698
Stroke	14.7	8.9	<0.001
COPD	4.2	1.8	<0.001
Creatinine (μmol/L)	93.00 (70.70, 125.70)	74.00 (62.00, 90.00)	<0.001
Hemoglobin (g/L)	128.00 (113.00, 143.00)	138.00 (125.00, 150.00)	<0.001
WBC (10^9^/L)	11.30 (8.72, 14.30)	9.54 (7.54, 11.98)	<0.001
K^+^(mmol/L)	4.00 (3.63, 4.46)	3.92 (3.64 ;4.21)	<0.001
Na^+^(mmol/L)	138.15 (135.50, 141.00)	139.20 (137.00, 141.70)	<0.001
Smoking status (%)			<0.001
Nonsmoker	62.4	43.9	
Ex-smoker	12.6	10.7	
Current smoker	24.9	45.4	

BMI: body mass index; SBP: systolic blood pressure; HR: heart rate; CAD: coronary artery disease; MI: myocardial infarction PCI: percutaneous coronary intervention; PAD: peripheral artery disease; COPD: chronic obstructive pulmonary disease; WBC: white blood cell.

Continuous variables are presented as median (interquartile range).

We included 5795 patients with NSTEMI and 17622 patients with STEMI in our study. Among patients with NSTEMI, 541 (9.3%) patients received early invasive approach. Among patients with STEMI, 7587 (43.0%) patients were treated with primary PCI, and 1739 (9.9%) patients received thrombolytic therapy.

### Independent predictors of in-hospital death

The association between baseline characteristics and in-hospital mortality are shown in Table 2. A total of 25 variables with P ≤ 0.25 were selected to fit the multivariable logistic regression model: age, BMI, systolic blood pressure, heart rate, creatinine level, red blood cell, white blood cell, serum potassium level, serum sodium level, sex, ST-elevation, anterior wall involvement, fatal arrhythmia, cardiac arrest, Killip classification, hypertension, hyperlipidemia, diabetes, prior CAD, MI, PCI, HF, stroke, COPD and smoking status. After stepwise selection, a total of 16 variables achieved a significance level of P ≤ 0.05 were identified as independent predictors of in-hospital death, including age, gender, BMI, SBP, heart rate, creatinine level, WBC count, serum potassium, serum sodium, ST-elevation on ECG, anterior wall involvement, cardiac arrest, Killip classification, medical history of hypertension, medical history of hyperlipidemia and smoking status (Table 3).

**Table 2:** Univariate analysis of the association between baseline characteristics and in-hospital mortality.

Variable	OR (95%CI)	P value
Age, per one year increase	1.066 (1.060, 1.072)	<0.0001
BMI, per 1 kg/m^2^ increase	0.911 (0.892, 0.930)	<0.0001
SBP, per 1 mmHg increase	0.979 (0.977, 0.982)	<0.0001
Heart rate, per 1 beat/min increase	1.022 (1.019, 1.025)	<0.0001
Creatinine level, per 1 μmol/Lincrease	1.006 (1.005, 1.006)	<0.0001
RBC, per 1 × 10^12^/L increase	0.984 (0.982, 0.986)	<0.0001
WBC, per 1 × 10^9^/L increase	1.107 (1.093, 1.122)	<0.0001
K^+^, per 1 mmol/L increase	1.557 (1.397, 1.736)	<0.0001
Na^+^, per 1 mmol/L increase	0.984 (0.978, 0.989)	<0.0001
Male	0.449 (0.397, 0.507)	<0.0001
ST-segment elevation	1.269 (1.109, 1.452)	<0.0001
Anterior wall involvement	1.510 (1.338, 1.703)	<0.0001
Fatal arrhythmia	2.704 (2.283, 3.203)	<0.0001
Cardiac arrest	6.412 (4.765, 8.628)	<0.0001
Killip classification		<0.0001
II vs. I	2.431 (2.086, 2.834)	
III vs. I	4.505 (3.670, 5.529)	
IV vs. I	12.41 (10.36, 14.86)	
Medical history		
Hypertension	1.244 (1.103, 1.404)	<0.0001
Hyperlipidemia	0.544 (0.406, 0.729)	<0.0001
Diabetes	1.255 (1.087, 1.448)	0.002
Coronary artery disease	0.419 (0.261, 0.673)	<0.0001
Myocardial infarction	1.226 (0.990, 1.519)	0.062
PCI	0.683 (0.493, 0.948)	0.023
Heart failure	3.002 (2.310, 3.903)	<0.0001
Stroke	1.733 (1.457, 2.063)	<0.0001
COPD	2.224 (1.621, 3.050)	<0.0001
Current smoker vs. non smoker	0.384 (0.334, 0.442)	<0.0001

BMI: body mass index; RBC: red blood cell; WBC: white blood cell; PCI: percutaneous coronary intervention; COPD; chronic obstructive pulmonary disease.

**Table 3:** Independent predictors of in-hospital death among AMI patients.

Variable	OR (95% CI)	P value
Age, per one year increase	1.053 (1.046, 1.060)	<0.0001
Cardiac arrest	3.218 (2.250, 4.601)	<0.0001
Killip classification		
II vs. I	1.440 (1.221, 1.699)	<0.0001
III vs. I	1.953 (1.554, 2.456)	<0.0001
IV vs. I	4.108 (3.327, 5.072)	<0.0001
Anterior wall involvement	1.404 (1.224, 1.611)	<0.0001
ST-segment elevation	1.397 (1.199, 1.628)	<0.0001
Hypertension	1.266 (1.103, 1.453)	<0.0001
Heart rate, per beat/min increase	1.013 (1.010, 1.016)	<0.0001
K^+^, per 1 mmol/L increase	1.264 (1.130, 1.414)	<0.0001
WBC, per 1 × 10^9^/L increase	1.075 (1.059, 1.091)	<0.0001
Cr, per μmol/L increase	1.003 (1.002, 1.004)	<0.0001
Na^+^, per 1 mmol/L increase	0.990 (0.982, 0.997)	0.006
SBP, per 1 mmHg increase	0.983 (0.980, 0.985)	<0.0001
BMI, per 1 kg/m^2^ increase	0.968 (0.946, 0.990)	0.004
Hyperlipidemia	0.726 (0.529, 0.995)	0.047
Current smoker vs. non smoker	0.702 (0.591, 0.835)	<0.0001
Male vs. Female	0.685 (0.585, 0.801)	<0.0001

BMI: Body mass index, SBP: Systolic blood pressure, Cr: Creatinine, WBC: white blood cell.

### CAMI risk score

**Figure 2:**
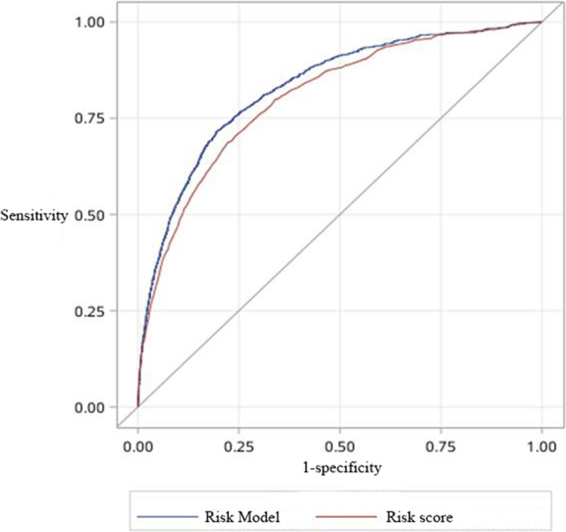
ROC curves of CAMI risk model and CAMI risk score within derivation cohort. Area under curve value was 0.83 (95% confidence interval (CI): 0.82 to 0.84) for CAMI risk model and 0.80 (95% CI: 0.77-0.82) for CAMI risk score.

**Figure 3:**
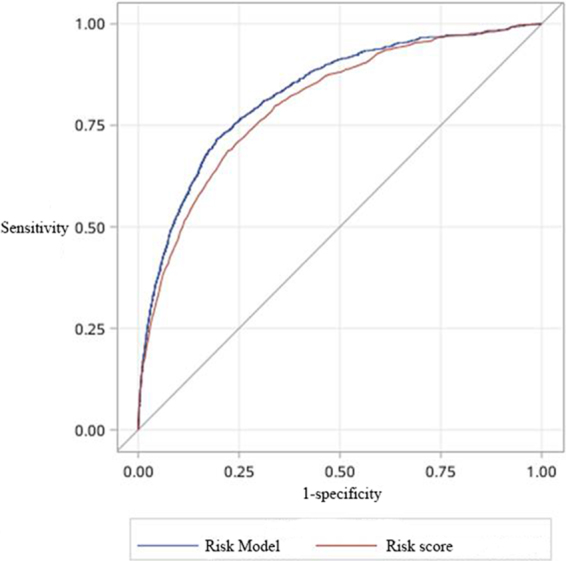
ROC curves of CAMI risk model and CAMI risk score within validation cohort. Area under curve value was 0.84 (95% confidence interval (CI): 0.82 to 0.86) for CAMI risk model and 0.80 (95% CI: 0.78–0.83) for CAMI risk score.

We developed a simplified risk score by attributing integer number to each variable according to their estimated coefficients (Table 4). Corresponding in-hospital mortality risk associated with each point is shown in supplementary Table 1. CAMI risk score ranges from 0 to 284, and corresponding in-hospital death risk ranges from 0.3% to 97.7%. Within derivation cohort, area under curve value for CAMI risk model was 0.83 (95% CI: 0.82-0.84). AUC value for the simplified CAMI risk score was only slightly worse than that of CAMI risk model (0.83 vs. 0.80, p = 0.07) (Fig. 2). Hosmer-Lemeshow P (HL-P) value for CAMI risk score was 0.10, which indicated good calibration. Within validation cohort, AUC value for CAMI risk model and score were 0.84 (95% CI: 0.82-0.86) and 0.80 (95% CI: 0.78-0.83), and no significant difference in AUC value was detected (P = 0.07) (Fig. 3).

**Table 4:** Scores attributed to each variable.

Variable	Level	Point	Variable	Level	Point
1. Age (years)	<55	0	9. Gender	Female	11
	[55–65)	18		Male	0
	[65–75)	33	10. ST-segment elevation		
	≥75	47		No	0
2. BMI (Kg/m^2^)	<18.5	11		Yes	10
	[18.5–24)	7	11. Anterior wall involvement		
	[24–28)	4		No	0
	>=28	0		Yes	10
3. SBP (mmHg)	<111	31	12. Cardiac arrest	No	0
	[111–128)	21		Yes	35
	[128–144)	13	13. Killip Classification		
	>=144	0		I	0
4. HR (bpm)	<66	0		II	11
	[66–76)	4		III	22
	[76–88)	8		IV	33
	>=88	15	14. Hyperlipidemia	No	10
5. Cr (μmol/L)	<63	0		Yes	0
	[63–75)	1	15. Smoking status		
	[75–90)	2	Nonsmoker		32
6. WBC (10^9^/L)	<7.67	0	Ex-smoker		21
	[7.67–9.61)	5	(quit smoking ≤ 1 year)		
	[9.61–12.08)	9	Ex-smoker		11
	≥12.08	17	(quit smokingå 1 year)		
7. K^+^ (mmol/L)	<3.66	0	Current smoker		0
	[3.66–3.92)	3	16. Na^+^(mmol/L)	<136.9	3
	[3.92–4.22)	4		[136.9–139.1)	2
	>=4.22	7		[139.1–141.3)	1
8. Hypertension	No	0		>=141.3	0
	Yes	7			

BMI: body mass index; SBP: systolic blood pressure; HR: heart rate.

We compared the diagnostic performance of CAMI risk score with GRACE risk score. The AUC value for CAMI score and GRACE risk score was 0.8043 and 0.8054 respectively (p = 0.8 for comparison). We also demonstrated that the diagnostic performance of CAMI risk score was superior to that of TIMI risk score (C-statistics: 0.8043 for CAMI and 0.7781 for TIMI risk score, p < 0.0001 for comparision).

Within derivation cohort, we divided all participants into three groups (Tertile I, II, III) based on tertiles. Each tertile contained approximately one third of the population (Table 5). Event rate increased significantly across tertiles: 1.12% in Tertile I (score range: 0–93), 3.47% in Tertile II (score range: 94–117), 14.70% in Tertile III (score range: ≥118). Within validation cohort, a similar pattern was observed and event rate also increased significantly across tertiles: 0.79% in Tertile I, 3.24% in Tertile II and 13.66% in Tertile III. Therefore, we defined Tertile I, II, III as low, intermediate and high risk group respectively.

**Table 5:** Event rate Across Different Risk Group.

	Low Risk Group (Tertile I)	Intermediate Risk Group (Tertile II)	High Risk Group (Tertile III)	P value
Score range	0–93	94–117	≥118	
**In-hospital mortality rate**				
Derivation Cohort	1.12% (64/5649)	3.47% (203/5843)	14.70% (883/6007)	<0.001
Validation Cohort	0.79% (15/1888)	3.24% (63/1945)	13.66% (276/2021)	<0.001

## Discussion

In a large-scale contemporary prospective registry of patients with AMI in China, we identified 16 independent predictors of in-hospital deaths, and by using these variables, we developed and validated a risk prediction tool of in-hospital death among AMI patients. CAMI risk score had high discrimination and calibration ability in both the derivation and validation cohort. A significant gradient of in-hospital mortality risk was identified with increased CAMI score.

### Comparison with GRACE

Many risk prediction tools have been developed to assess short- or long-term mortality risk of ACS patients, among which GRACE and TIMI risk score were the most popular and validated tool. We demonstrated that CAMI risk score was non-inferior to GRACE and was superior to TIMI risk score in terms of c-statistics. In this part, we focused our comparison with GRACE risk score because CAMI score shared similar study design with GRACE score (GRACE score was designed from registry data while TIMI score was derived from clinical trial data).

Since the creation of GRACE risk score, patient profile of AMI has changed over time, with a slight increase in NSTEMI and a decrease in STEMI, and an overall decline in AMI^15^. Updated diagnostic criteria of AMI also have an impact on the detection and prevalence of AMI. For instance, the introduction of troponin, a relatively new biomarker, into AMI definition was reported to lead to increased annual incidence rate^16^. A proportion of unstable angina (UA) patients will be diagnosed as MI in the era of high-sensitive troponin, leading to an increase in MI and a reciprocal decrease in UA^6^. Due to improvement in medication and invasive treatment, in-hospital mortality of STEMI has declined significantly^17^. These changes require periodic updates of existing models, which justifies our work.

Although GRACE registry was a large-scale multinational registry enrolling patients from 14 countries in North America, South America, Europe, Australia, and New Zealand, few participants were from Asia^18^. However, including participants from Asia is of great clinical significance: The population of Asia is greater than 4.2 million, which accounts for around 60% world population^19^. ACS is the leading cause of mortality in Asia and is estimated to account for half of the global burden^20^. Our work bridged the evidence gap, developed and validated a novel risk model by using data from CAMI registry, the largest prospective multicenter registry of patients with AMI in Asia region.

### Variables in the model

Many variables in CAMI risk model were identical to those in GRACE risk score including: age, SBP, creatinine, ST-segment elevation, cardiac arrest, Killip classification, hypertension, anterior wall involvement. Novel variables in our score included: hyperlipidemia, gender, BMI and smoking. Unexpectedly, hyperlipidemia was a protective factor of in-hospital death. This may be explained by the fact that patients with hyperlipidemia are more likely to take lipid-lowering medications including statins, and there is a significant increase trend in statins use over decades^21^, which maybe associated with improved prognosis. Our study found that female gender was an independent risk factor of in-hospital death, while in GRACE risk score, gender was not associated with mortality risk in multivariable analysis. This discrepancy maybe caused by difference in study population. Approximately one third participants in GRACE registry were diagnosed with UA^22^, while we did not include UA patients. Compared with MI, patients with UA were more often females, and had lower death risk since they do not have myocardial necrosis. These factors can have an impact on the association between gender and mortality^6^.

Our study demonstrated the phenomenon of smoker’s and obesity paradox. Although smoking and obesity are well established risk factors of coronary artery disease, current smokers (compared with nonsmokers) and patients with high BMI (compared with patients with low BMI) paradoxically had lower in-hospital mortality. Possible explanations for smoker’s paradox were difference in fibrinolytic therapy effectiveness among smokers vs. nonsmokers. Smokers had increased level of circulating fibrinogen and more fibrin-rich thrombus compared with nonsmokers. Therefore, smokers may have improved myocardial perfusion and prognosis after fibrinolysis treatment^23^. The phenomenon of obesity paradox had been found in cases of several heart conditions including acute myocardial infarction, hypertension, heart failure, atrial fibrillation^24^. Several mechanisms have been proposed to explain obesity paradox: Obese individuals may have higher energy reserve in response to acute stress^25^, and more likely to receive optimal medication therapy and invasive treatment^26^. Of note, we included smoking status and BMI in CAMI risk score to improve the diagnostic performance of the risk model, and should not interpreted as the encouragement of smoking or obesity.

### Limitations

First, CAMI risk score should be further validated in separate large-scale cohort. Second, all participants were from China, whether CAMI score can be applied to other ethnicities need further validation. CAMI score was designed for rapid risk assessment after presentation, so we didn’t include some laboratory test variables such as troponin level and left ventricular ejection fraction.

## Conclusions

Using data from a large-scale contemporary cohort, we developed and validated a risk score to accurately predict risk of in-hospital death risk among patients with AMI. CAMI risk score had high discrimination and calibration ability and is likely to be useful for clinicians to assess in-hospital death risk accurately and to select optimal management.

## Electronic supplementary material

Supplementary Information

## References

[1] Murray CJ (2015). Global, regional, and national disability-adjusted life years (DALYs) for 306 diseases and injuries and healthy life expectancy (HALE) for 188 countries, 1990–2013: quantifying the epidemiological transition. Lancet (London, England).

[2] Members WG (2016). Heart Disease and Stroke Statistics-2016 Update: A Report From the American Heart Association. Circulation.

[3] Townsend N (2016). Cardiovascular disease in Europe 2016: an epidemiological update. European Heart Journal.

[4] Wei-Wei C (2017). China cardiovascular diseases report 2015: a summary. Journal of geriatric cardiology: JGC.

[5] Amsterdam EA, Wenger NK (2015). The 2014 American College of Cardiology ACC/American Heart Association guideline for the management of patients with non-ST-elevation acute coronary syndromes: ten contemporary recommendations to aid clinicians in optimizing patient outcomes. Circulation.

[6] Damman P (2017). 2015 ESC guidelines for the management of acute coronary syndromes in patients presenting without persistent ST-segment elevation: comments from the Dutch ACS working group. Netherlands Heart Journal.

[7] Morrow DA (2000). TIMI risk score for ST-elevation myocardial infarction: A convenient, bedside, clinical score for risk assessment at presentation: An intravenous nPA for treatment of infarcting myocardium early II trial substudy. Circulation.

[8] Addala S (2004). Predicting mortality in patients with ST-elevation myocardial infarction treated with primary percutaneous coronary intervention (PAMI risk score). American Journal of Cardiology.

[9] Granger CB (2004). Predictors of hospital mortality in the global registry of acute coronary events. Acc Current Journal Review.

[10] De LG (2004). Prognostic assessment of patients with acute myocardial infarction treated with primary angioplasty: implications for early discharge. Circulation.

[11] Halkin A (2005). Prediction of mortality after primary percutaneous coronary intervention for acute myocardial infarction: the CADILLAC risk score. Journal of the American College of Cardiology.

[12] Xu H (2015). The China Acute Myocardial Infarction (CAMI) Registry: A national long-term registry-research-education integrated platform for exploring acute myocardial infarction in China. American Heart Journal.

[13] Thygesen K (2012). Third universal definition of myocardial infarction. Global Heart.

[14] Sullivan LM, Massaro JM, D’Agostino RB (2004). Presentation of multivariate data for clinical use: The Framingham Study risk score functions. Statistics in medicine.

[15] Yeh RW (2010). Population trends in the incidence and outcomes of acute myocardial infarction. New England Journal of Medicine.

[16] Agüero, F. *et al*. New myocardial infarction definition affects incidence, mortality, hospitalization rates and prognosis. *European Journal of Preventive Cardiology* (2014). 10.1177/2047487314546988 25139771

[17] Physicians, A. C. O. E. & Al, E. 2013 ACCF/AHA guideline for the management of ST-elevation myocardial infarction: a report of the American College of Cardiology Foundation/American… - PubMed - NCBI.

[18] Investigators G (2001). Rationale and design of the GRACE (Global Registry of Acute Coronary Events) Project: a multinational registry of patients hospitalized with acute coronary syndromes. American Heart Journal.

[19] Chan MY (2016). Acute coronary syndrome in the Asia-Pacific region. International Journal of Cardiology.

[20] Ohira T, Iso H (2013). Cardiovascular Disease Epidemiology in Asia. Circulation Journal Official Journal of the Japanese Circulation Society.

[21] Salami, J. A. *et al*. National Trends in Statin Use and Expenditures in the US Adult Population From 2002 to 2013: Insights From the Medical Expenditure Panel Survey. *Jama Cardiology* **2** (2016). 10.1001/jamacardio.2016.4700 27842171

[22] Fox KA (2002). Management of acute coronary syndromes. Variations in practice and outcome; findings from the Global Registry of Acute Coronary Events (GRACE). European heart journal.

[23] Aune E, Røislien J, Mathisen M, Thelle DS, Otterstad JE (2011). The “smoker’s paradox” in patients with acute coronary syndrome: a systematic review. Bmc Medicine.

[24] Lavie CJ, Mcauley PA, Church TS, Milani RV, Blair SN (2014). Obesity and Cardiovascular Diseases: Implications Regarding Fitness, Fatness, and Severity in the Obesity Paradox. Journal of the American College of Cardiology.

[25] Banack HR, Kaufman JS (2014). The obesity paradox: understanding the effect of obesity on mortality among individuals with cardiovascular disease. Preventive Medicine.

[26] Niedziela J (2014). The obesity paradox in acute coronary syndrome: a meta-analysis. European Journal of Epidemiology.

