# Supplementary material for: The CAMI-score: A Novel Tool derived From CAMI Registry to Predict In-hospital Death among Acute Myocardial Infarction Patients
Source: Sci Rep. 2018 Jun 13;8:9082. doi: 10.1038/s41598-018-26861-z (PMC5998057; doi:10.1038/s41598-018-26861-z)
Supplement: Supplementary file 1 — Supplementary Information [file 41598_2018_26861_MOESM1_ESM.docx]

**The CAMI-score: A Novel Tool derived From CAMI Registry to Predict In-hospital Death among Acute Myocardial Infarction Patients**

**Chenxi Song, MD1, Rui Fu, MD1, Kefei Dou, MD, PhD1※, Jingang Yang, MD, PhD1, Haiyan Xu, MD, PhD1, Xiaojin Gao, MD, PhD1, Wei Li, PhD1, Guofeng Gao, MD1, Zhiyong Zhao, MD1, Jia Liu, PhD1, Yuejin Yang, MD, PhD1※**

**Supplementary material**

**Supplementary table 1 In-hospital mortality risk associated with each point**

| **0** | **0.003163666** | **46** | **0.014602462** | **92** | **0.064715199** |
| --- | --- | --- | --- | --- | --- |
| **1** | **0.003271092** | **47** | **0.015092423** | **93** | **0.066772668** |
| **2** | **0.003382153** | **48** | **0.015598563** | **94** | **0.068890731** |
| **3** | **0.003496971** | **49** | **0.016121399** | **95** | **0.071070863** |
| **4** | **0.003615673** | **50** | **0.016661463** | **96** | **0.073314557** |
| **5** | **0.003738389** | **51** | **0.017219303** | **97** | **0.075623316** |
| **6** | **0.003865255** | **52** | **0.017795481** | **98** | **0.077998662** |
| **7** | **0.003996408** | **53** | **0.018390578** | **99** | **0.080442125** |
| **8** | **0.004131992** | **54** | **0.019005191** | **100** | **0.082955247** |
| **9** | **0.004272158** | **55** | **0.019639934** | **101** | **0.08553958** |
| **10** | **0.004417056** | **56** | **0.020295437** | **102** | **0.088196679** |
| **11** | **0.004566847** | **57** | **0.02097235** | **103** | **0.090928109** |
| **12** | **0.004721693** | **58** | **0.02167134** | **104** | **0.093735434** |
| **13** | **0.004881764** | **59** | **0.022393095** | **105** | **0.09662022** |
| **14** | **0.005047233** | **60** | **0.023138319** | **106** | **0.099584033** |
| **15** | **0.005218283** | **61** | **0.023907737** | **107** | **0.102628432** |
| **16** | **0.005395097** | **62** | **0.024702093** | **108** | **0.105754971** |
| **17** | **0.005577869** | **63** | **0.025522152** | **109** | **0.108965192** |
| **18** | **0.005766797** | **64** | **0.0263687** | **110** | **0.112260628** |
| **19** | **0.005962086** | **65** | **0.027242542** | **111** | **0.115642792** |
| **20** | **0.006163947** | **66** | **0.028144505** | **112** | **0.119113182** |
| **21** | **0.006372598** | **67** | **0.029075438** | **113** | **0.12267327** |
| **22** | **0.006588266** | **68** | **0.030036213** | **114** | **0.126324504** |
| **23** | **0.006811183** | **69** | **0.03102772** | **115** | **0.130068301** |
| **24** | **0.007041588** | **70** | **0.032050876** | **116** | **0.133906045** |
| **25** | **0.007279731** | **71** | **0.033106619** | **117** | **0.137839082** |
| **26** | **0.007525866** | **72** | **0.034195909** | **118** | **0.141868717** |
| **27** | **0.007780259** | **73** | **0.03531973** | **119** | **0.145996206** |
| **28** | **0.00804318** | **74** | **0.036479089** | **120** | **0.150222758** |
| **29** | **0.008314913** | **75** | **0.037675018** | **121** | **0.154549525** |
| **30** | **0.008595746** | **76** | **0.03890857** | **122** | **0.158977598** |
| **31** | **0.008885978** | **77** | **0.040180826** | **123** | **0.163508005** |
| **32** | **0.00918592** | **78** | **0.041492886** | **124** | **0.168141705** |
| **33** | **0.009495889** | **79** | **0.042845877** | **125** | **0.172879582** |
| **34** | **0.009816215** | **80** | **0.04424095** | **126** | **0.177722439** |
| **35** | **0.010147235** | **81** | **0.04567928** | **127** | **0.182670998** |
| **36** | **0.010489299** | **82** | **0.047162064** | **128** | **0.187725888** |
| **37** | **0.010842768** | **83** | **0.048690524** | **129** | **0.192887647** |
| **38** | **0.011208013** | **84** | **0.050265906** | **130** | **0.198156711** |
| **39** | **0.011585418** | **85** | **0.05188948** | **131** | **0.203533412** |
| **40** | **0.011975377** | **86** | **0.053562537** | **132** | **0.209017973** |
| **41** | **0.012378297** | **87** | **0.055286393** | **133** | **0.214610503** |
| **42** | **0.012794599** | **88** | **0.057062384** | **134** | **0.220310989** |
| **43** | **0.013224714** | **89** | **0.05889187** | **135** | **0.226119298** |
| **44** | **0.013669088** | **90** | **0.06077623** | **136** | **0.232035167** |
| **45** | **0.014128179** | **91** | **0.062716866** | **137** | **0.238058199** |
| **138** | **0.244187861** | **186** | **0.617309748** | **234** | **0.889549235** |
| **139** | **0.25042348** | **187** | **0.625192022** | **235** | **0.892797957** |
| **140** | **0.256764239** | **188** | **0.633008462** | **236** | **0.8959623** |
| **141** | **0.26320917** | **189** | **0.640755561** | **237** | **0.8990438** |
| **142** | **0.269757158** | **190** | **0.64842995** | **238** | **0.902044009** |
| **143** | **0.276406932** | **191** | **0.656028403** | **239** | **0.904964482** |
| **144** | **0.283157068** | **192** | **0.663547844** | **240** | **0.907806783** |
| **145** | **0.290005981** | **193** | **0.670985344** | **241** | **0.910572477** |
| **146** | **0.296951929** | **194** | **0.678338134** | **242** | **0.913263131** |
| **147** | **0.303993009** | **195** | **0.685603598** | **243** | **0.915880308** |
| **148** | **0.311127159** | **196** | **0.69277928** | **244** | **0.918425569** |
| **149** | **0.318352153** | **197** | **0.699862881** | **245** | **0.920900468** |
| **150** | **0.325665609** | **198** | **0.706852268** | **246** | **0.92330655** |
| **151** | **0.333064983** | **199** | **0.713745463** | **247** | **0.925645353** |
| **152** | **0.340547574** | **200** | **0.720540652** | **248** | **0.927918401** |
| **153** | **0.348110525** | **201** | **0.72723618** | **249** | **0.930127206** |
| **154** | **0.355750828** | **202** | **0.733830549** | **250** | **0.932273267** |
| **155** | **0.363465324** | **203** | **0.740322418** | **251** | **0.934358066** |
| **156** | **0.371250708** | **204** | **0.746710603** | **252** | **0.936383068** |
| **157** | **0.379103534** | **205** | **0.752994068** | **253** | **0.938349723** |
| **158** | **0.387020222** | **206** | **0.759171931** | **254** | **0.94025946** |
| **159** | **0.394997059** | **207** | **0.76524345** | **255** | **0.942113688** |
| **160** | **0.403030207** | **208** | **0.771208031** | **256** | **0.943913797** |
| **161** | **0.411115711** | **209** | **0.777065216** | **257** | **0.945661157** |
| **162** | **0.419249506** | **210** | **0.782814682** | **258** | **0.947357113** |
| **163** | **0.427427422** | **211** | **0.788456236** | **259** | **0.949002993** |
| **164** | **0.435645195** | **212** | **0.793989811** | **260** | **0.950600097** |
| **165** | **0.443898475** | **213** | **0.799415463** | **261** | **0.952149705** |
| **166** | **0.452182833** | **214** | **0.804733363** | **262** | **0.953653075** |
| **167** | **0.460493771** | **215** | **0.809943793** | **263** | **0.955111438** |
| **168** | **0.468826736** | **216** | **0.815047144** | **264** | **0.956526004** |
| **169** | **0.477177121** | **217** | **0.820043908** | **265** | **0.957897957** |
| **170** | **0.485540283** | **218** | **0.824934673** | **266** | **0.959228461** |
| **171** | **0.493911551** | **219** | **0.82972012** | **267** | **0.96051865** |
| **172** | **0.502286234** | **220** | **0.834401014** | **268** | **0.96176964** |
| **173** | **0.510659635** | **221** | **0.838978205** | **269** | **0.962982519** |
| **174** | **0.519027058** | **222** | **0.843452618** | **270** | **0.964158353** |
| **175** | **0.527383821** | **223** | **0.847825249** | **271** | **0.965298183** |
| **176** | **0.535725268** | **224** | **0.852097162** | **272** | **0.966403027** |
| **177** | **0.544046775** | **225** | **0.856269482** | **273** | **0.967473881** |
| **178** | **0.552343763** | **226** | **0.860343393** | **274** | **0.968511715** |
| **179** | **0.560611706** | **227** | **0.864320129** | **275** | **0.969517477** |
| **180** | **0.568846144** | **228** | **0.868200975** | **276** | **0.970492093** |
| **181** | **0.577042691** | **229** | **0.871987257** | **277** | **0.971436466** |
| **182** | **0.58519704** | **230** | **0.875680344** | **278** | **0.972351476** |
| **183** | **0.59330498** | **231** | **0.879281637** | **279** | **0.973237982** |
| **184** | **0.601362397** | **232** | **0.882792571** | **280** | **0.974096821** |
| **185** | **0.609365283** | **233** | **0.886214609** | **281** | **0.974928808** |

| **282** | **0.975734738** | **283** | **0.976515385** | **284** | **0.977271503** |
| --- | --- | --- | --- | --- | --- |

**Table 2 Missing data for each baseline variable**

| **Baseline characteristics** | **In-hospital death** | **In-hospital survival** |
| --- | --- | --- |
| **Age** | 1504 (0) | 21913 (0) |
| **Gender** | 1504 (0) | 21913 (0) |
| **Body mass index** | 1504 (0) | 21913 (0) |
| **Chest pain** | 1218 (286) | 20073 (1840) |
| **ST-segment elevation** | 1483 (21) | 21514 (399) |
| **Anterior wall involvement** | 1500 (4) | 21732 (181) |
| **Systolic blood pressure** | 1470 (34) | 21768 (145) |
| **Heart rate** | 1497 (7) | 21797(116) |
| **Fatal arrhythmia** | 1495 (9) | 21808 (105) |
| **Cardiac arrest** | 1498 (6) | 21825 (88) |
| **Killip classification** | 1490 (14) | 21754 (159) |
| **Hypertension** | 1500 (4) | 21853 (60) |
| **Hyperlipidemia** | 1502 (2) | 21834 (79) |
| **Diabetes** | 1498 (6) | 21829 (84) |
| **Premature family CAD** | 1500 (4) | 21811 (102) |
| **Prior myocardial infarction** | 1502 (2) | 21802 (111) |
| **Prior PCI** | 1502 (2) | 21761 (152) |
| **Prior CABG** | 1501 (3) | 21786 (127) |
| **Heart failure** | 1501 (3) | 21790 (123) |
| **Peripheral artery disease** | 1501 (3) | 21779 (134) |
| **Stroke** | 1499 (5) | 21795 (118) |
| **COPD** | 1489 (15) | 21679 (234) |
| **Creatinine** | 1300 (204) | 20610 (1303) |
| **Hemoglobin** | 1381 (123) | 21446 (467) |
| **WBC** | 1382 (122) | 21469 (444) |
| **K^+^** | 1399 (105) | 21520 (393) |
| **Na^+^** | 1400 (104) | 21506 (407) |
| **Smoking status** | 1494 (10) | 21790 (123) |

The number in the brackets denotes number of patients with missing data.

**Table 3 An example CRF in CAMI registry**

| 1. Baseline Characteristics | |
| --- | --- |
| 1.01 Ethnicity □ Han □ Non-Han □ Foreigner □ Other | |
| 1.02 Height □□□cm | |
| 1.03 Weight □□□Kg | |
| 1.04 Marital status □Married □Divorced □Unmarried □Unclear | |
| 1.05 Medical insurance □Basic medical care □ New rural cooperative medical system | |
| 1.06 Education level □Illiteracy □Primary school □High school □Colledge | |
| 1.07 Occupation _________ | |
| 1.08 Living status □Living alone □Living with others | |
| 1. Onset characteristics | |
| 2.01 Place of the attack □Home □Public area □Hospital □ Working place | |
| 2.02 Time of the attack □□□□year □□month□□day□□hour□□minute □Unclear | |
| 2.03 Symptoms □Chest pain □ Atypical symptoms □Unclear | |
| 2.04 Symptom onset □Sudden and lasting onset □Intermittent onset | |
| 2.05 Duration of symptom □□hours □□ minutes □Unclear | |
| - 1. Chest pain location □Anterior part of chest □Chest and chin □Left arm   □Upper abdomen □Mandible □Left shoulder and both forearms  □ Back pain □Other location | |
| 2.07 Atypical symptoms □Shortness of breathe □Syncope □ Cardiac arrest □Unclear | |
| 2.08 Accompanying symptoms □Diaphoresis □Nausea □Syncope | |
| 2.09 Cardiac arrest prior to admission □Yes □No □Unclear | |
| 1. Presentation characteristics | |
| 3.01 Admission to hospital □Emergency system □ Transferred from other hospitals  □By other family members □On one’s own | |
| 3.02 Thrombolysis in ambulance □Yes □No □Unclear | |
| 3.021 Pre-hospital defibrillation □Yes □No □Unclear | |
| 3.03 Thrombolysis at other hospitals □Yes □No □Unclear | |
| 3.04 Primary PCI at other hospitals □Yes □No □Unclear | |
| 3.05 Pre-hospital ECG □Yes □No □Unclear | |
| 3.06 Time to hospital □□□□year □□month□□day□□hour□□minute □Unclear | |
| 4 Electrocardiography (ECG) | |
| 4.01 Time of first ECG □□□□year □□month□□day□□hour□□minute □Unclear  □Not performed | |
| 4.02 Rhythm □Sinus □Atrial fibrillation □Pacing rhythm □Ventricular fibrillation  □Atrioventricular block □Ventricular tachycardia □Other | |
| 4.03 ECG characteristics  □ST-segment elevation □ST-segment depression  □ Complete left bundle branch block □Complete right bundle branch block  □Non-specific ST-T segment change □Abnormal Q wave  □No change □Other □Not performed | |
| 4.03 AMI location based on ECG  □Anteroseptal wall (V_1_,V_2_) □Anterior wall(V_1_-V_3,4_) □Extensive anterior wall (V_1_-V_5,6_)  □High lateral wall (I,avL) □Anterior lateral wall (V_5_,V_6_) □Posterior wall (II,III,avF)  □Posterior wall (V_7_-V_9_) □ Right ventricular □Difficult to locate | |
| Cadiac enzyme and biomarker | |
| 4.05 CK-MB peak level ______□IU/L □ng/ml □Not performed | |
| 4.06 Troponin □TNI (to 4.07) □TNT (to 4.08) □hs-Troponin □Not performed | |
| 4.07 TNI peak level □□□ng/ml | |
| 4.08 TNT peak level □□□ng/ml | |
| 4.09 hs-Troponin peal level □□□ng/ml | |
| 5.0 Diagnosis and treatment | |
| 5.01 Diagnosis □STEMI □NSTEMI | |
| Initial Evaluation | |
| 5.02 Heart rate □□□beat/min | |
| 5.03 Systolic blood pressure □□□mmHg □not performed □not detected | |
| 5.04 Diastolic blood pressure □□□mmHg □not performed □not detected | |
| 5.05 Heart failure □Yes □No □Uncertain | |
| 5.06 Cardiac shock □Yes □No □Uncertain | |
| 5.07 Killip classification □I □II □III □IV | |
| Medication at emergency department | |
| 5.08 Medication at at emergency department □Yes (to 5.09-5.11) □No (to 5.12) | |
| 5.09 Medication  □Dopamine  □Dobutamine  □Adrenaline  □Metaraminol □Norepinephrine  □None of the above | |
| 5.10 Administration method □Bolus injection □ Intravenous drip | |
| 5.11 Administration duration □□□hours | |
| 5.12 Defibrillation □Yes □No | |
| 6. Risk factor and medical history | |
| 6.01 Hypertension □Yes □No □Unclear | |
| 6.02 Treatment □Yes □No □Unclear | |
| 6.03 Hyperlipidemia □Yes □No □Unclear | |
| 6.04 Type □Hypercholesterolemia □Hypertriglyceridemia □Both □Unclear | |
| 6.05 Statins treatment □Yes □No □Unclear | |
| 6.06 Type II diabetes □Yes (to 6.07-6.08) □No □Unclear | |
| 6.07 Date of first diabetes diagnosis  □□□□year □□month□□day□□hour□□minute □Unclear | |
| 6.08 Management □Diet □Oral medication □Insulin □DPP-4/GLP-1 □Unclear | |
| 6.09 Smoking □Current smoker □Ex-smoker (quit smoking more than one year)  □Ex-smoker (quit smoking less than one year) □Non-smoker | |
| 6.10 Premature coronary artery disease □Yes □No □Unclear | |
| 6.11 Menstrual history □Menopause □Not Menopause □Unclear | |
| Medical history | |
| 6.12 Coronary artery disease □Yes □No | |
| 6.13 Prior coronary lesion with DS%＞50% □Yes □No □Unclear | |
| 6.14 Prior angina □Yes □No □Unclear | |
| 6.15 Prior myocardial infarction □Yes (to 6.16-6.19) □No □Unclear | |
| 6.16 Number of prior heart attacks:____ | |
| 6.17 MI location □ Anterior wall □Non-anterior wall □Both □Unclear | |
| 6.18 Date of first MI □□ years ago □Unclear | |
| 6.19 Emergent revascularization □Thrombolysis □Primary PCI □ Not reperfusion □Unclear | |
| 6.20 Elective PCI □Yes (to 6.21-6.23) □No □Unclear | |
| 6.21 Number of prior PCI: ____ | |
| 6.22 Date of last PCI procedure: □□□□year □□month□□day □Unclear | |
| 6.23 Number of stents: ____ | |
| 6.24 Prior CABG: □Yes (to 6.25) □No □Unclear | |
| 6.25 Date of last CABG: □□□□year □□month□□day □Unclear | |
| 6.26 Prior heart failure: □Yes □No □Unclear | |
| 6.27 Prior atrial fibrillation: □Yes □No □Unclear | |
| 6.28 Prior stroke: □Yes (to 6.29) □No □Unclear | |
| 6.29 Type of stroke □Cerebral hemorrhage □Cerebral infarction □Unclear | |
| 6.30 Peripheral artery disease □Yes □No □Unclear | |
| 6.31 Prior aortic disease □Yes (to 6.32) □No □Unclear | |
| 6.32 Type of aortic disease □Aortic dissection □Aortic aneurysm □Other  □Aortic hematoma □Aortic stenosis | |
| 6.33 Renal dysfunction □Yes (to 6.34) □No □Unclear | |
| 6.34 Dialysis □Yes □No □Unclear | |
| 6.35 COPD □Yes □No □Unclear | |
| 6.36 Immune system disease □Yes □No □Unclear | |
| 6.37 Malignant tumor □Yes (to 6.38) □No □Unclear | |
| 6.38 Type of malignant tumor:____ | |
| 6.39 Thyroid disease □Yes (to 6.40) □No □Unclear | |
| 6.40 Type of thyroid disease: □Hyperthyroidism □Hypothyroidism □Thyroiditis □Other | |
| 7. STEMI emergent reperfusion | |
| Thrombolysis | |
| 7.01 Thrombolysis □Yes (to 7.02-7.05) □No (to 7.21) | |
| 7.02 Time of thrombolysis □□□□year □□month□□day□□minute □Unclear | |
| 7.03 Time of thrombolysis from disease onset  □less than 3 hours □3-6 hours □6-12 hours □12-24 hours □1-3 days | |
| 7.04 Medication  □Streptokinase □Urokinase □rtPA □Staphylokinase □Reteplase □Other | |
| 7.041Dose _____mg □IU | |
| 7.042 Thrombolytic effect □Successful □Unsuccessful □Unclear | |
| 7.05 Treatment after thrombolysis □Medication □Rescue PCI □Transfer to superior hospital | |
| Emergent coronary angiography | |
| 7.06 Coronary angiography □Yes □No | |
| 7.061 Dominant type □Right dominant □ Left dominant □Balance type | |
| 7.062 Lesion location | |
| 7.063 Coronary angiography □One-vessel □Two vessel □Three vessel | |
| 7.0631 LM lesions □Yes □No | |
| 7.0632 Infarct-related coronary artery  □LAD □LM □PDA □LCX □RCA □Dia □PLA □OM □Bypass artery □ Unclear | |
| 7.0633 Most severe degree of stenosis: _____% | |
| 7.0634 TIMI flow □0 □I □II □III | |
| 7.0635 Thrombus □Primary thrombus □Thrombus in stent □No | |
| Primary PCI | |
| 7.07 Primary PCI □Yes (to 7.08-15, 7.18,7.20) □No (to 7.22) | |
| 7.08 PCI type □Primary PCI □Rescue PCI □Coronary angiography or PCI after thrombolysis | |
| 7.09 Anticoagulants  □Heparin □Low molecular weight heparin □Bivalirudin □Other | |
| 7.10 GP IIb/IIIa □Yes □No | |
| 7.11 PCI sequence  □Emergency department→Cath Lab→Ward □Emergency department→Ward→Cath Lab  □Ambulance→Cath Lab→Ward □AMI during hospitalization→Cath Lab  □Out-patient→Ward→Cath Lab | |
| 7.12 Time of PCI from onset  □＜3 hours □3-6 hours □6-12 hours □12-24 hours □ 1-3 days □Other | |
| 7.13 Time of entering Cath lab □□□□year □□month□□day□□hour □Unclear | |
| 7.14 PCI approach □Radial artery □Femoral artery □Ulnar artery □Brachial artery | |
| PCI treatment of infarct-related artery | |
| 7.15 PCI treatment of infarct-related artery □Yes (to 7.16) □No | |
| 7.16 Time of balloon dilation  □□□□year □□month□□day□□hour□□minute □Unclear | |
| 7.17 PCI method □Thrombus aspiration □PTCA □Stent | |
| 7.171 Stent type □DES □BMS □Both | |
| 7.172 BMS stent number:____ | |
| 7.173 DES stent number: ____ | |
| 7.174 TIMI flow grade post PCI □0 □I □II □III | |
| 7.175 Complication □Yes (to 7.176) □No | |
| 7.176 Complication  □No reflow □Acute in-stent restenosis □Coronary artery perforation □Emergent CABG  □Cardiac arrest □Death □Slow blood flow □Coronary artery rupture  □Cardiac tamponade □Ventricular tachycardia □Other | |
| PCI treatment of non-infarct related artery | |
| 7.18 PCI treatment of non-infarct related artery □Yes (to 7.19-7.198) □No (to 7.20) | |
| 7.19 Reason  □Severe stenosis □Based on experience □ Suspected myocardial ischemia □Other | |
| 7.191 Artery segment treated: | |
| 7.192 Stent implantation: □Yes □No | |
| 7.193 Stent type □DES □BMS □Both | |
| 7.194 BMS stent number:____ | |
| 7.195 DES stent number: ____ | |
| 7.196 TIMI flow grade post PCI □0 □I □II □III | |
| 7.197Complication □Yes (to 7.176) □No | |
| 7.198 Complication  □No reflow □Acute in-stent restenosis □Coronary artery perforation □Emergent CABG  □Cardiac arrest □Death □Slow blood flow □Coronary artery rupture  □Cardiac tamponade □Ventricular tachycardia □Other | |
| Reperfusion efficiency | |
| 7.20 ST-segment resolution □＜50% □＞50% □Close to equipotential line | |
| 7.21 Reason not to perform thrombolysis | |
| 7.22 Reason not to perform primary PCI | |
| Emergent CABG | |
| 7.23 Emergent CABG | |
| 7.23 Emergent CABG □Yes (to 7.26) □No (to 8.0) | |
| 7.24 In-hospital outcome □Survive □Died | |
| 8.0 NSTEMI emergent reperfusion | |
| 8.01 Emergent coronary angiography □Yes □No | |
| 8.011 Dominant type □Right dominant □ Left dominant □Balance type | |
| 8.012 Lesion location | |
| 8.013 Coronary angiography □One-vessel □Two vessel □Three vessel | |
| 8.014 LM lesions □Yes □No | |
| 8.015 Infarct-related coronary artery  □LAD □LM □PDA □LCX □RCA □Dia □PLA □OM □Bypass artery □ Unclear | |
| 8.016 Most severe degree of stenosis: _____% | |
| 8.017 TIMI flow □0 □I □II □III | |
| 8.018 Thrombus □Primary thrombus □Thrombus in stent □No | |
| Primary PCI | |
| 8.02 Primary PCI □Yes (to 7.08-15, 7.18,7.20) □No (to 7.22) | |
| 8.03 Indication for primary PCI □Persistent myocardial ischemia □Instable hemodynamics  □Instable cardiac electrical activity □Cardiac dysfunction □Other | |
| 8.04 Time of emergent PCI □□□□year □□month□□day□□hour□□minute □Unclear | |
| 8.05 PCI approach □Radial artery □Femoral artery □Ulnar artery □Brachial artery | |
| 8.06 Anticoagulants  □Heparin □Low molecular weight heparin □Bivalirudin □Other | |
| 8.07 GP IIb/IIIa □Yes □No | |
| PCI treatment of infarct-related artery | |
| 8.08 PCI treatment of infarct-related artery □Yes (to 8.10) □No (to 8.09) | |
| 8.09 If no, the reason  □TIMI flow grade 3 □Contradiction for PCI □Severe condition □Patient rejection | |
| 8.10 PCI method □Thrombus aspiration □PTCA □Stent | |
| 8.101 Stent type □DES □BMS □Both | |
| 8.102 BMS stent number:____ | |
| 8.103 DES stent number: ____ | |
| 8.104 TIMI flow grade post PCI □0 □I □II □III | |
| 8.105 Complication □Yes (to 7.176) □No | |
| 8.106 Complication  □No reflow □Acute in-stent restenosis □Coronary artery perforation □Emergent CABG  □Cardiac arrest □Death □Slow blood flow □Coronary artery rupture  □Cardiac tamponade □Ventricular tachycardia □Other | |
| PCI treatment of non-infarct related artery | |
| 8.11 PCI treatment of non-infarct related artery □Yes (to 8.12) □No | |
| 8.12 Reason  □Severe stenosis □Based on experience □ Suspected myocardial ischemia □Other | |
| 8.121 Artery segment treated: | |
| 8.122 Stent type □DES □BMS □Both | |
| 8.123 BMS stent number:____ | |
| 8.124 DES stent number: ____ | |
| 8.125 TIMI flow grade post PCI □0 □I □II □III | |
| 8.126 Complication □Yes (to 8.127) □No | |
| 8.127 Complication  □No reflow □Acute in-stent restenosis □Coronary artery perforation □Emergent CABG  □Cardiac arrest □Death □Slow blood flow □Coronary artery rupture  □Cardiac tamponade □Ventricular tachycardia □Other | |
| Emergent CABG | |
| 8.13 Emergent CABG □Yes (to 8.14) □No (to 9.0) | |
| 8.14 In-hospital outcome □Survive □Died | |
| 9. Medication | |
| 9.01 Aspirin loading dose (300mg) □Yes □No | |
| 9.02 Aspirin maintenance dose □Yes □No | |
| 9.03 Thienopyridine □Clopidogrel (9.031-9.032) □Ticagrelor (9.033-9.034) □Prasugrel □ Sarpogrelate □Not take | |
| 9.031 Loading dose □300mg □600mg □No loading dose | |
| 9.032 Routine dose □150mg □75mg □50mg | |
| 9.033 Loading dose □180mg □No loading dose | |
| 9.034 Routine dose □90mg bid □90mg qd | |
| 9.04 Other antiplatelet □Cilostazol □Dipyridamole □Not clear □None | |
| 9.05 GPIIb/IIIa antagonist □Yes (9.06) □No (9.07) | |
| 9.06 Duration □＜24h □24-48h □＞48h | |
| 9.07  Heparin □Yes □No | |
| 9.08 Low-weight heparin □Yes □No | |
| 9.09 Fondaparinux sodium □Yes □No | |
| 9.10 Oral anticoagulants □Yes(9.11-9.12) □No (9.13) | |
| 9.11 Medication type □Warfarin □Shaaban □Dabigatran □Other | |
| 9.13 Loading dose of statins within 24h □Yes □No | |
| 9.14 Maintain statins treatment □Yes (9.15-9.16) □No (9.17) | |
| 9.15 Statins type □Simvastatin □Atorvastatin □Pravastatin □Lovastatin □ Other | |
| 9.16 Dose ___mg/d | |
| 9.17 Nitrate Esters □Yes □No | |
| 9.18 β-blocker □Intravenous Injection  □oral □Intravenous Injection and oral Intravenous Injection □No | |
| 9.19 Ca^2+^ blocker □Yes □No | |
| 9.20 ACEI/ARB □Yes □No | |
| 9.21 Antiarrhythmic medication □Yes (9.22) □No (9.23) | |
| 9.22 Type  □Amiodarone  □Lidocaine □ Propafenone  □Other | |
| 9.23 Aldosterone antagonist □Yes □No | |
| 9.24 Diuretic □Yes □No | |
| 9.25 Non-statins lipid-lowering agents □Yes □No | |
| 9.26 Gastric acid inhibitor □Yes (to 9.27) □No (to 9.28) | |
| 9.27 Type □PPI inhibitor □H2 receptor blocker | |
| 9.28 Oral Chinese medicine □Yes (to 9.29) □No (to 9.30) | |
| 9.29 Type □Danshen □Tonxinluo □Shexiang □Other | |
| 9.30 Intravenous Chinese medicine □Yes (to 9.31) □No (to 10.0) | |
| 9.31 Type_______ | |
| 10 Blood biomarker and ultrasound | |
| 10.1 Fasting glucose ______mmol/L □Not done | |
| 10.011 OGTT 2-hour glucose _____mmol/L □Not done | |
| 10.02 Creatinine _____mmol/L □Not done | |
| 10.03 Hemoglobin _____g/L □Not done | |
| 10.04 Triglyceride ______mmol/L □Not done | |
| 10.05 LDL-c ______mmol/L □Not done | |
| 10.06 HDL-c ______mmol/L □Not done | |
| 10.07 K^＋^______mmol/L □Not done | |
| 10.08 Hs-CRP ______mmol/L □Not done | |
| 10.09 HBA1C ______% □Not done | |
| 10.10 BNP ______ □pg/ml □ng/ml □pg/L □ng/L □pmol/L | □Not done |
| 10.11 NT-ProBNP ______ □pg/ml □ng/ml □pg/L □ng/L □pmol/L | □Not done |
| 10.12 Thyroid function □Done (10.121) □Not done | |
| 10.121 TSH______□IU/ml □mIU/L; TT4______□g/dl □nmol/L;  TT3______□ng/ml □ng/dl□nmol/L; FT4______□pg/ml □ng/dl □pmol/L;  FT3______□pg/ml □ng/dl □pmol/L | |
| 10.122 CK ______U/L □Not done | |
| 10.123 MYO ______ng/ml □Not done | |
| 10.124 ALT ______U/L □Not done | |
| 10.125 AST ______U/L □Not done | |
| 10.126 Scr ______μmol /L □Not done | |
| 10.127 TBil ______μmol /L □Not done | |
| 10.128 DBil ______μmol /L □Not done | |
| 10.129 IBil ______μmol /L □Not done | |
| Cardiac Ultrasound | |
| 10.13 Date of first cardiac ultrasound □□□□year □□month □□day □Not done | |
| 10.14 LVEDd □□mm | |
| 10.15 LVEF% □□% | |
| 10.16 Wall motion abnormality □Hypokinesis □Akinesis □Dyskinesis □No | |
| 11. Elective coronary angiography | |
| 11.01 Elective coronary angiography □Yes (to 11.02) □No (to 12.0) | |
| 11.02 First angiography □ First angiography (to CAG1C) □Second angiography (to 11.03) | |
| 11.03 Re-angiography  □The same as first angiography □Patent stent □In-stent thrombus □In-stent occlusion | |
| Coronary angiography | |
| 11.021 Dominant type □Right dominant □ Left dominant □Balance type | |
| 11.022 Lesion location | |
| 11.023 Coronary angiography □One-vessel □Two vessel □Three vessel | |
| 11.024 LM lesions □Yes □No | |
| 11.025 Infarct-related coronary artery  □LAD □LM □PDA □LCX □RCA □Dia □PLA □OM □Bypass artery □ Unclear | |
| 11.026 Most severe degree of stenosis: _____% | |
| 11.027 TIMI flow □0 □I □II □III | |
| 11.028 Thrombus □Primary thrombus □Thrombus in stent □No | |
| 11.04 Elective PCI □Yes (to 11.05) □No | |
| 11.05 Date □□□□year □□month□□date □□unclear | |
| 11.06 Time of PCI from onset  □1-3 days □3-7 days □7-14 days □14-28 days □more than 28 days | |
| 11.07 Indication for elective PCI □Myocardial ischemia □Infarct-related artery PCI  □Non-infarct related artery PCI □Other | |
| 11.08 PCI approach □Radial artery □Femoral artery □Ulnar artery □Brachial artery | |
| 11.09 Anticoagulants  □Heparin □Low molecular weight heparin □Bivalirudin □Other | |
| 11.10 GP IIb/IIIa □Yes □No | |
| 11.101 Vessel segment treated | |
| 11.102 Stent type □DES □BMS □Both | |
| 11.103 Number of BMS □□ | |
| 11.104 Number of DES □□ | |
| 11.105 Final blood flow post PCI □0 □1 □2 □3 | |
| 11.106 Complication □ Yes □No | |
| Selective CABG | |
| 11.11 Selective CABG □Yes (to 11.12) □No (to 12.0) | |
| 11.12 In-hospital outcome □Died □Survival | |
| 12. Major complication and adverse events during hospitalization | |
| 12.01 CABG during hospitalization □ Yes □No | |
| 12.02 Cardiac arrest □ Yes □No | |
| 12.03 Heart failure □ Yes □No | |
| 12.04 Cardiac shock □ Yes □No | |
| 12.05 Mechanical complication □ Yes (to 12.06-12.07) □No | |
| 12.06 Type □Ventricular septal rupture □Papillary muscle rupture □Free ventricular wall rupture □False ventricular aneurysm | |
| 12.07 Treatment □Interventional device closure □Surgery □ Intervention procedure failure and surgery □ No treatment | |
| 12.08 Severe arrhythmia □Yes (12.09-11) □No | |
| 12.09 Type □Ventricular tachycardia □Severe bradycardia  □ AV block second degree type II  □AVB (third degree ) □AVB ( high degree ) | |
| 12.10 Special treatment □Temporary pacemaker □Permanent pacemaker □ICD □CRT □Other | |
| 12.11 Electric defibrillation □Yes □No | |
| 12.12 Recurrent myocardial ischemia □Yes □No | |
| 12.13 Re-infarction □Yes (12.14-12.17) □No | |
| 12.14 Causes for re-infarction | |
| 12.15 Re-emergent revascularization □Yes □No | |
| 12.16 Original lesion □Yes □No | |
| 12.17 In-stent thrombus □Yes □No | |
| 12.18 IABP □Yes(to 12.19) □No | |
| 12.19 Time of application  □Before PCI □ During PCI □ Post PCI □The patient didn’t receive PCI | |
| 12.20 Other specific treatment □No □Ventilator  □Left ventricular assist device □ECMO | |
| 12.21 Stroke □Yes (12.22-12.23) □No | |
| 12.22 Date □□□□year□□month□□day □Unclear | |
| 12.23 Type □Cerebral Hemorrhage □Cerebral Hemorrhage after infarction  □Cerebral infarction □Unclear | |
| 12.24 Other hemorrhage events □Yes □No | |
| 12.29 Pulmonary embolism □Yes □No | |
| 12.30 Peripheral artery embolism □Yes (to 12.31) □No | |
| 12.31 Location ______ | |
| 12.32 Allergy □Contrast medium allergy □Other medication allergy □No | |
| 12.33 Death □Yes (to 12.34-12.35) □No (to 13.0) | |
| 12.34 Date of death | |
| 12.35 Cause of death □Cardiac death □Cardiac shock □Heart failure □Fatal arrhymia  □Mechanical complication □Intervention complication □Multiorgan dysfunction □ Cerebral hemorrhage □Pulmonary infection □Cerebral infarction □Allergy □Major bleeding □Other | |
| 13 Discharge | |
| 13.01 Please select the following:  □Discharge after vital signs are stable □Transfer to surgical department to receive CABG  □automatic discharge □Transfer to superior hospital □Transfer to other rehabilitation medical constitution □Transfer to other department (in the same department) | |
| 13.02 Aspirin □Yes □No | |
| 13.03 Clopidogrel □Yes (13.04-13.05) □No | |
| 13.04 Type □Imported □Domestic | |
| 13.05 Dose ____mg/d | |
| 13.06 Ticagrelor □Yes □No | |
| 13.07 β-blocker □Yes (to 13.08-13.09) □No | |
| 13.08 Type □Metoprolol □Metoprolol sustained-release tablets □Bisoprolol □Carvedilol | |
| 13.09 Dose ____mg/d | |
| 13.10 Statins □Yes (to 13.11-13.12) □No | |
| 13.11 Type □Simvastatin □Atorvastatin □Pravastatin □Lovastatin □ Other | |
| 13.12 Dose ____mg/d | |
| 13.13 ACEI/ARB □Yes (to 13.14-13.15) □No | |
| 13.14 Type ____ | |
| 13.15 Dose ____mg/d | |
| 13.16 Anti-arrhythmia □Yes (to 13.17-13.18) □No | |
| 13.17 Type □Propafenone □Mexiletine □Amiodarone □Sotalol □Other | |
| 13.18 Dose ____mg/d | |
| 13.19 Digoxin □Yes □No | |
| 13.20 Spironolactone □Yes □No | |
| 13.21 Chinese medication □Yes (13.22) □No | |
| 13.22 Type □Tongxinluo □Danshen □Musk pill □Other | |
| 13.23 Gastric acid inhibitor □Yes (13.24) □No (13.25) | |
| 13.24 Type □PPI □H2 receptor inhibitor | |
| 13.25 Glucose-lowering treatment □Yes (13.26) □No (to 14) | |
| 13.26 Oral medication □Sulfonylurea □Biguanides □Glucosidase  □Thiazolidinedione □Insulin secretagogues □No | |
| 13.27 Insulin □Yes (dose:____unit) □No | |
| 13.28 Other □DDP-4 inhibitor □GLP-1 | |
| 14. Hospitalization days and cost | |
| 14.01 Hospitalized for □□days | |
| 14.02 Intensive care unit for □□days | |
| 14.03 Emergency department □□hours | |
| 14.04 Emergency cost: ____________yuan | |
| 14.05 Hospitalization cost: ____________yuan | |
| 14.06 Material cost: ____________yuan | |
| 14.07 Examination cost: ____________yuan | |
| 14.08 Surgery cost: ____________yuan | |
| 14.09 Medication cost: ____________yuan | |
| 15. 01 AMI classification prior to discharge: □I □II □III □Iva □IVb □IVc □V | |
| 15.02 The patient participated in other clinical study □Yes (to 15.03) □No | |
| 15.03 Type: ____________ | |
